# Potential Role of Bone Scintigraphy in the Diagnosis of Calciphylaxis

**DOI:** 10.1055/s-0043-1760760

**Published:** 2024-01-29

**Authors:** Khushboo Gupta, Pokhraj Prakashchandra Suthar, Neetal Bhave, Jagadeesh S. Singh, Sindhuja M. K. Venkatraman, Rahul B. Jadhav

**Affiliations:** 1 Department of Radiology, Nuclear Medicine Division, University of Arkansas for Medical Sciences, Little Rock, Arkansas, United States; 2 Department of Radiology and Nuclear Medicine, Rush University Medical Center, Chicago, Illinois, United States; 3 Department of Pathology, Rush University Medical Center, Chicago, Illinois, United States; 4 Department of Radiology, Neurointervention Radiology, University of Arkansas for Medical Sciences, Little Rock, Arkansas, United States

**Keywords:** calciphylaxis, ^99m^ Tc MDP bone scan, computed tomography, ESRD, histopathology

## Abstract

Nonosseous abnormalities are often seen on bone scans and can be related to a wide variety of pathology ranging across vascular, infection, and inflammatory etiology. Diffuse soft tissue radiotracer uptake on bone scans is typically attributed to renal or metabolic derangements. Calciphylaxis is the deposition of calcium in small blood vessels, skin, and other organs leading to vascular obstruction and skin necrosis. It is a rare disorder with unknown pathophysiology. Diagnosis of calciphylaxis is challenging and requires an interdisciplinary approach including clinical findings, laboratory results, medical imaging, and skin biopsy. An early diagnosis is important as the disease is associated with high morbidity and mortality. The purpose of this review article is to highlight the role of bone scintigraphy in the evaluation of calciphylaxis and to correlate the findings with other imaging modalities and histopathology.

## Introduction

Calciphylaxis is an uncommon disease. It results from calcification of the medial layer of small arteries causing luminal narrowing and occlusion, which may lead to the formation of blood clots, necrosis, and ulceration of the overlying skin. 1 Serious secondary infections and sepsis may follow which also form the primary cause of mortality in these patients. There are multiple etiological factors associated, predominantly renal failure and hyperparathyroidism. 2 The disease was first recognized by Bryant and White in patients with uremia. 3 Since then, the incidence is increasingly recognized in patients with end-stage renal disease (ESRD) on dialysis. The diagnosis has, however, been a challenge due to the coexistence of common features with other disease processes such as cellulitis, coumarin necrosis, peripheral vascular disease, polyarteritis nodosa, etc. The associated mortality rate remains high, approximately 60 to 80%, due to poor healing rates and secondary infections. 4 Identification of the disease highly relies on clinical symptoms, risk factors, radiographic and nuclear medicine findings along with skin biopsy. Extraosseous calcium deposition in vascular structures and skin ulceration may demonstrate increased uptake of Tc-99m methylene diphosphonate ( ^99m^ Tc MDP) on whole-body bone scintigraphy. This characteristic feature may help in the diagnosis and determine the extent of the disease. Computed tomography (CT) demonstrates macroscopic calcification of vascular structures and soft tissues. In this case-based review article, we try to highlight the approach to the diagnosis of calciphylaxis with an emphasis on bone scintigraphy features.

## Cases

### Case 1

**Fig. 1:**
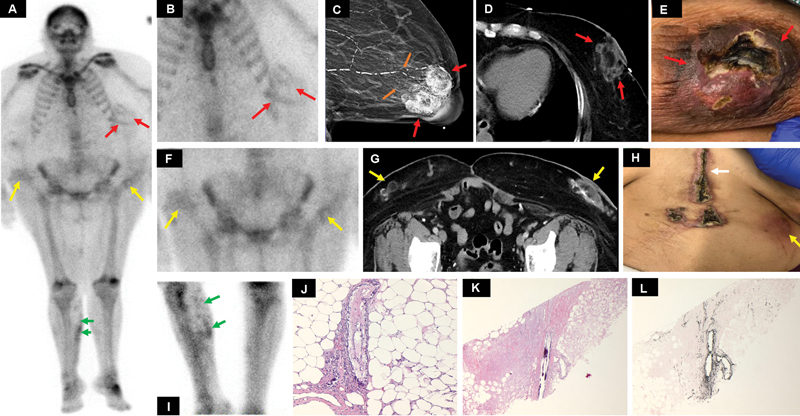
( **A** ) Anterior image of the whole body and ( **B** , **F** , **I** ) delayed static triple-phase bone scan images demonstrating moderate tracer retention in the soft tissues involving the left breast ( *red arrows* in ( **A** ) and ( **B** )), lateral aspect of the lower abdominal wall ( *yellow arrows* in ( **A** ) and ( **F** )), and subcutaneous tissue of the right calf ( *green arrows* in ( **A** ) and ( **I** )). ( **C** ) Mammography of the left breast demonstrated subareolar mass and thickened left nipple-areolar complex ( *red arrows* ) along with benign calcification ( *solid orange* arrows). ( **D** ) Axial computed tomography (CT) through the lower chest demonstrated thick-walled peripherally enhancing subcutaneous abscess with trace complex fluid and fat stranding in the inferior left breast ( *red arrows* ). ( **G** ) Axial CT through the abdomen demonstrates multiple anterior abdominal wall subcutaneous calcified lesions with fat stranding ( *yellow arrows* ). ( **E** , **H** ) The clinical picture of subcutaneous wounds involving the left breast ( *red arrows* in ( **E** )) and abdominal wall ( *yellow arrow* ( **H** )) along with an anterior central abdominal hernia repair scar ( *white arrow* ( **H** )). ( **J** ) Histopathology of the left breast tissue shows a blood vessel in the breast parenchyma with calcification of the vessel wall (hematoxylin and eosin stain, 100× magnification). ( **K** ) Histopathology of the left breast tissue shows a fragment of breast parenchyma with necrosis and acute inflammation adjacent to a blood vessel with a calcified wall (hematoxylin and eosin stain, 40× magnification). ( **L** ) A von Kossa calcium stain highlighting the calcifications in the vessel wall, suggestive of calciphylaxis (von Kossa calcium stain, 40× magnification).

A 61-year-old female with a history of ESRD presented with complaints of painful breast and lower abdominal subcutaneous lumps ( Fig. 1E,H ). CT chest, abdomen, and pelvis were advised which demonstrated soft tissue nodules with peripheral calcifications in the lower abdomen and pelvic wall ( Fig. 1G ). There was a heterogeneous fat density area with stranding in the retroareolar region of the left breast ( Fig. 1D ). A similar finding with mild fat stranding was seen in the right breast soft tissue. Mammography examination of the bilateral breast demonstrated subareolar soft tissue density in the bilateral breast with vascular calcifications in the left breast and thickening of the left nipple-areolar complex ( Fig. 1C ). The patient underwent a triple-phase bone scan, performed after administration of 24.8 mCi of ^99m^ Tc MDP, which demonstrated diffuse tracer retention in the soft tissues with nonvisualization of the kidneys, consistent with renal failure status ( Fig. 1A ). In the delayed phase there was persistent moderate-grade tracer uptake in the left breast soft tissue ( Fig. 1B ) and diffuse tracer uptake along the subcutaneous nodules in the lateral wall of the lower abdomen and pelvis ( Fig. 1F ). Additionally, there was irregular moderate tracer uptake along the medial right calf subcutaneous tissue ( Fig. 1I ). Biopsy from the left breast soft tissue showed fragments of breast parenchyma with necrosis and acute inflammation adjacent to the blood vessels, with calcification on hematoxylin and eosin stain ( Fig. 1J,K ) as well as a von Kossa stain ( Fig. 1L ). Calciphylaxis was considered a clinical diagnosis and the patient was treated with thiosulfates.

### Case 2

**Fig. 2:**
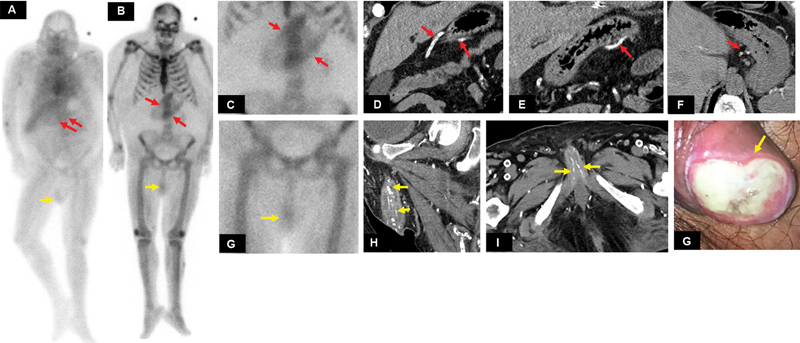
( **A** ) Anterior image of the whole-body blood pool, ( **B** ) anterior whole-body 3-hour delayed image, as well as ( **C** ) static triple-phase bone scan images of the upper abdomen and ( **G** ) pelvis demonstrate diffuse increased tracer uptake along the stomach walls ( *red arrows* in ( **A** – **C** )) and the external genitalia (penile region, *yellow arrow* in ( **A** , **B** , and **G** )), respectively. ( **D** ) Coronal, ( **E** ), and ( **F** ) axial noncontrast computed tomography (CT) images through the upper abdomen, demonstrate extensive vascular calcifications ( *red arrows* ). ( **G** ) Clinical picture of the penile ulcer ( *yellow arrow* ). ( **H** ) Coronal and ( **I** ) axial CT images through the pelvis demonstrate extensive penile vascular calcifications ( *yellow arrows* ).

A 51-year-old male with a history of ESRD presented with a 1-month history of scrotal swelling and pain. Clinically, there was an ulcerated penile lesion with discoloration ( Fig. 2G ). A triple-phase bone scan performed after administration of 26.1 mCi of ^99m^ Tc MDP demonstrated irregular diffuse increased tracer uptake in the upper abdominal region as well as in the region of external genitalia on early vascular ( Fig. 2A,B ) and delayed static bone phase ( Fig. 2C,G ). There was diffuse soft tissue retention of tracer activity with poor visualization of small-sized bilateral kidneys indicative of renal insufficiency on whole-body bone scan ( Fig. 2B ). CT of the abdomen pelvis demonstrated extensive vascular calcification along the lesser curvature of the stomach ( Fig. 2D–F ) and penile region ( Fig. 2H,I ), concerning for visceral calciphylaxis.

### Case 3

**Fig. 3:**
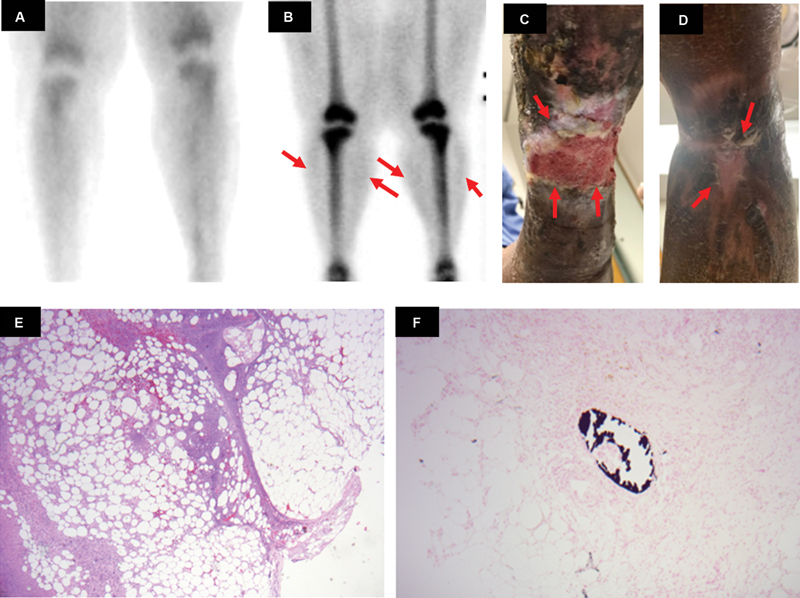
( **A** ) Anterior blood pool static triple-phase bone scan image of bilateral lower extremities demonstrates no significant tracer uptake in the subcutaneous soft tissue. ( **B** ) Anterior delayed static bone scan image of bilateral lower extremities demonstrates minimal diffuse tracer activity along the subcutaneous soft tissue ( *red arrows* ). ( **C** and **D** ) Clinical picture of bilateral lower limbs demonstrating extensive ulceration ( *red arrows* ). ( **E** ) Hematoxylin and eosin stain (10× magnification) of skin biopsy of the lesion showed necrosis of fibroadipose tissue, hemorrhage, and a mixed inflammatory infiltrate. ( **F** ) von Kossa stain (40× magnification) of skin biopsy of the lesion showed calcification of small vessel, confirming the diagnosis of extremity calciphylaxis.

A 61-year-old female with a history of ESRD presented with chronic bilateral leg pain and extensive lower extremity wounds. Clinically, there was hyperpigmentation, erythema, and ulcerated wounds in the bilateral distal lower limbs ( Fig. 3C,D ). A triple-phase bone scan performed with intravenous administration of 25.1 mCi of ^99m^ Tc MDP showed mildly increased tracer uptake in the soft tissues of the lower extremities on delayed images ( Fig. 3B ) without any significant increase in vascularity in the early soft tissue phase ( Fig. 3A ). Skin biopsy of the lesion in hematoxylin and eosin stain ( Fig. 3E ) showed necrosis of the fibroadipose tissue, hemorrhage, and mixed inflammatory infiltrate. Calcification of small vessel media was evident on the von Kossa stain, confirming the diagnosis of extremity calciphylaxis ( Fig. 3F ).

### Case 4

**Fig. 4:**
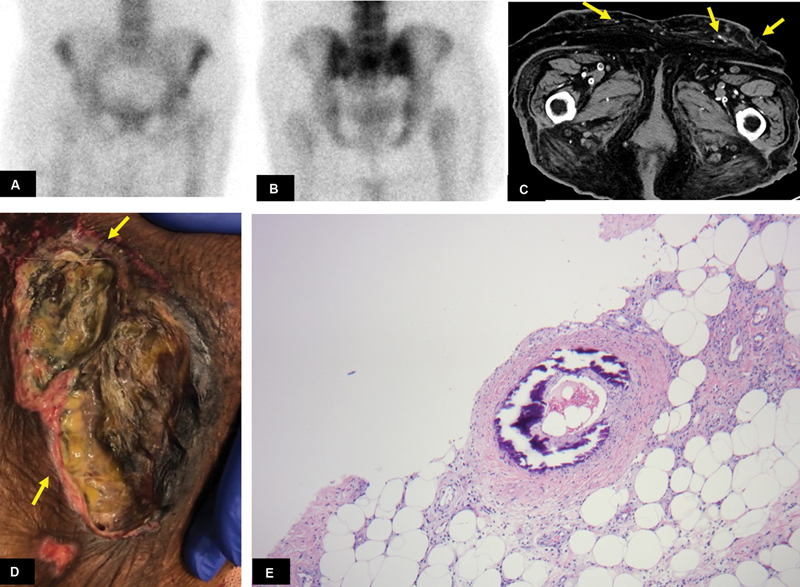
( **A** ) Anterior and ( **B** ) posterior delayed 3-hour static bone scan images of the lower abdomen and pelvis demonstrated no abnormal increased tracer uptake along the subcutaneous tissue. ( **C** ) Noncontrast computed tomography (CT) axial image through the lower abdomen pelvis demonstrates subcutaneous soft tissue calcification, thickening with stranding, and ulceration in the anterior abdominal wall, concerning for calciphylaxis ( *yellow arrows* ). ( **D** ) Clinical image of left lower quadrant irregular marginated skin ulceration with black eschar formation ( *yellow arrows* ). ( **E** ) Hematoxylin and eosin stain (40× magnification) of skin biopsy of the lesion shows soft tissue with fat necrosis and concentric calcification of media of the small vessel, suggestive of calciphylaxis.

A 69-year-old female with a history of type IV renal tubular acidosis and ESRD presented with a 1-month history of abdominal pain. Clinically, there was progressive worsening of the left lower quadrant with irregular skin ulceration with black eschar formation ( Fig. 4D ). The patient underwent a bone scan, performed after intravenous administration of 23.3 mCi of ^99m^ Tc MDP, which showed no significant abnormal tracer uptake in the lower abdomen and pelvis soft tissue ( Fig. 4A,B ). CT of the abdomen and pelvis demonstrated subcutaneous soft tissue calcification, thickening with stranding in the flank region, presumed to be related to truncal calciphylaxis ( Fig. 4C ). Skin biopsy of the lesion showed soft tissue with fat necrosis and concentric calcification of media of the small vessel ( Fig. 4E ), suggestive of calciphylaxis.

### Case 5

**Fig. 5:**
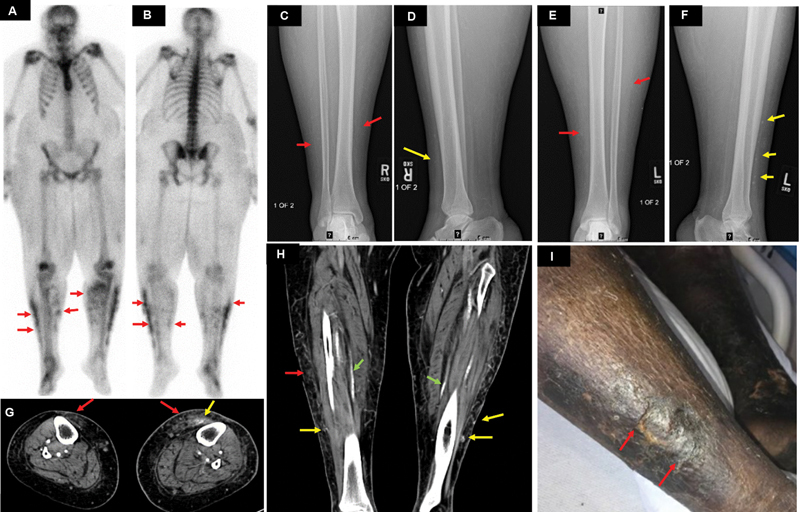
( **A** ) Anterior and ( **B** ) posterior delayed whole-body bone scan images demonstrate irregular increased patchy tracer uptake along the subcutaneous tissue of bilateral distal lower limbs ( *red arrows* ). ( **C** ) Frontal and ( **D** ) lateral radiographs of the right distal lower limb as well as ( **E** ) and ( **F** ) frontal and lateral radiographs of the left distal lower limb, respectively, demonstrate diffuse lower leg soft tissue swelling with vascular calcifications ( *red arrows* ) with additional punctate soft tissue calcifications also seen along the lower legs ( *yellow arrows* ). ( **G** ) Axial and ( **H** ) coronal images of the contrast-enhanced computed tomography (CT) of bilateral lower limbs demonstrate extensive atherosclerotic arterial calcifications bilaterally ( *green arrows* in ( **H** )), along with soft tissue swelling ( *red arrows* ) and punctate calcifications ( *yellow arrows* ), concerning for extremity calciphylaxis. ( **I** ) Clinical image of bilateral lower limbs demonstrating subcutaneous ulcers with hyperpigmentation and erythema.

A 53-year-old female with a history of ESRD presented with worsening bilateral lower extremity pain. Clinically, there was hyperpigmentation and erythema of the distal lower limbs ( Fig. 5I ). Plain radiographs showed diffuse bilateral lower leg soft tissue swelling with vascular calcifications ( Fig. 5C–F ). A whole-body bone scan, performed after intravenous administration of 26.3 mCi of ^99m^ Tc MDP, demonstrated moderate diffuse radiopharmaceutical uptake in the subcutaneous soft tissue of bilateral lower limbs ( Fig. 5A and B ). Nonvisualization of kidneys related to renal failure status was also noted. CT of the lower extremities demonstrated subcutaneous soft tissue stranding and calcifications, more in the left lower limb, concerning extremity calciphylaxis ( Fig. 5G and H ).

## Discussion

Calciphylaxis is more prevalent in females, predominantly of Caucasian ethnicity. 5 Multiple risk factors are associated with calciphylaxis, including obesity, diabetes mellitus, immunosuppression, warfarin treatment, hypoalbuminemia, ESRD patients on prolonged dialysis, and hyperparathyroidism. 6 Dysregulation of calcium, phosphorus, and parathyroid hormone levels results in vascular calcification involving tunica media or intima along with the secondary formation of calcified atherosclerotic plaque in vessel walls. Vascular smooth muscle cells (VSMCs), osteoprotegerin (OPG), and bone morphogenetic protein-4 (BMP-4) get involved at the cellular level and lead to the initiation of the vascular calcification process. 7

Hypercoagulability in calciphylaxis is related to an imbalance between prothrombotic and antithrombotic responses as well as a reduction in the expression of protein C and S receptors. The development of thrombosis evolves into tissue necrosis and ulceration. Clinically, patients present with painful subcutaneous indurated nodules and plaques limited to dermis and hypodermis. As the disease progresses, there is severe pain with the formation of nonhealing stellate shape ulceration and black eschar. Secondary infection of ulcerated lesions of calciphylaxis is common in adipose-rich areas like the trunk and lower extremities. Proximal lesions in the lower abdomen and proximal thigh are associated with a worse prognosis, and it occurs in 44 to 68% of patients. 8 9

### Diagnostic Modalities

The biopsy is the current gold standard for the diagnosis of calciphylaxis. Vascular and extravascular soft tissue calcification, necrosis, panniculitis, and local inflammation are the hallmarks of histopathology of skin biopsy. 10 11 12 Perieccrine calcification is highly specific for the diagnosis of calciphylaxis. However, in absence of calcification in the specimen, calciphylaxis cannot be excluded and skin biopsy may lack sensitivity in such cases. There are risks associated with biopsy which include bleeding, induction of necrosis, superimposed infection, and formation and propagation of new lesions. Other limiting factors include difficult sites to access biopsy samples, for example, visceral calciphylaxis in the lungs, gastrointestinal tract, and brain. 13

For the above reasons, a skin biopsy is often avoided, and diagnosis usually relies on other clinical tests and medical imaging. Laboratory tests including serum calcium, serum phosphorus, vitamin D, parathyroid level, and renal function test are typically performed along with inflammation markers consisting of serum C-reactive protein (CRP) and erythrocyte sedimentation rate (ESR), complete blood count, and blood culture. Evaluation of coagulation profile, exclusion of autoimmune disease, and malignancy additionally contribute to the disease assessment. 14

The role of radiology in the diagnosis of calciphylaxis is not well established. A plain radiograph may demonstrate vascular calcification in dermal and subcutaneous tissue in a net-like and arborization pattern. However, in patients with ESRD calcification is common and not specific for the diagnosis of calciphylaxis. CT scans show signs of soft tissue and vascular calcification, and features of inflammation with better resolution. Whole-body screening is optional with CT, but with limitations of high radiation exposure. The study performed on 10 patients with calciphylaxis by Bonchak et al demonstrated vascular calcification in 8 patients on CT. 15

Triple-phase nuclear bone scintigraphy with ^99m^ Tc MDP may have an important role in the diagnosis of calciphylaxis. 7 The avidity of MDP ligand to extraskeletal calcified tissues is known and can be used to study the disease process. Typical features of a triple-phase bone scan include increased flow and pooling of tracer activity in the initial phase with increased uptake in the delayed phase in the regions involved with calciphylaxis. Soft tissue and visceral calcification can be seen with bone scintigraphy. 16 The extent of the disease involvement can be studied on the whole-body image, which is associated with less radiation exposure as compared with CT and is quintessential in patients with renal failure. Bone scan performed with tomography and low-end CT (single-photon emission computerized tomography [SPECT]-CT) helps in localizing the lesions. In a study of 36 patients by Fine and Zacharias, bone scan was positive in 97% of the patients with calciphylaxis. 17 Serial bone scan is used to monitor disease progression and response to treatment. Compared with a plain radiograph or CT, bone scintigraphy with SPECT-CT assesses the exact anatomical location of active disease and the extent of the disease.

Although there are no proven therapies for calciphylaxis, patients may benefit from a normalization of calcium and phosphate levels, and meticulous wound care to prevent secondary infection. From all perspectives, early diagnosis is the key component of managing this complex disease. In this review, we would like to focus on the role of bone scintigraphy in the clinical diagnosis of calciphylaxis and discuss interesting representative cases from our institution.

## Conclusion

Calciphylaxis is a disease of vasculopathy caused by a variety of etiological factors. Laboratory blood analysis plays a limited role in diagnosis formation. Imaging modalities offer a better direction and mainly include radiographs, CT scans, and bone scintigraphy. CT imaging demonstrates calcification of the involved area while bone scintigraphy demonstrates three-phase positive soft tissue uptake in most cases. There were rare instances of biopsy-proven calciphylaxis lesions with mild to no accountable tracer uptake, perhaps related to the diminished flow of tracer activity due to calcified and occluded blood vessels or the presence of a high degree of fibrotic/necrotic tissue. However, the reason remains largely unknown and amenable to deeper research in pathophysiology. Overall, a bone scan helps in assessing the active disease, and extent of the disease, tracking the response to treatment, and determining the region of biopsy, which is usually considered to exclude patients with characteristic lesions without underlying renal dysfunction. As more insight is gained into this debilitating disease, there is expected improvement in optimizing the treatment management and prevention of disease progression.
